# Evaluating Nursing Workloads and Optimizing Staffing for Severe Pneumonia in Elderly under DRGs

**DOI:** 10.1093/geroni/igaf122.2423

**Published:** 2025-12-31

**Authors:** Rong Zhang, Yanhong Wang, Fei Xie

**Affiliations:** Shanxi Bethune Hospital, Taiyuan, Shanxi, China (People’s Republic); Shanxi Bethune Hospital, Taiyuan, Shanxi, China (People’s Republic); Shanxi Bethune Hospital, Taiyuan, Shanxi, China (People’s Republic)

## Abstract

**Objective:**

This study evaluates nursing workload and human resource challenges for elderly severe pneumonia patients under DRG payment, proposing strategies to balance care quality and cost efficiency.

**Methods:**

A mixed-method design was applied in a tertiary hospital (2023-2024).Nursing Activities Score(NAS) and DRG data quantified workload for 150 elderly patients.Regression analysis identified workload drivers(age, comorbidities, hospital stay).Interviews assessed allocation challenges.

**Results:**

The findings revealed that the mean NAS score for elderly patients with severe pneumonia was 78.5, substantially exceeding that of non-severe cases, thereby indicating a considerable nursing workload. The DRG grouping data demonstrated that nursing costs accounted for 35% of the total medical expenses associated with severe pneumonia, with direct nursing expenditures representing the highest proportion.Multiple regression analysis identified patient age, number of comorbidities, and length of hospital stay as the principal factors influencing nursing workload (P < 0.05).Several critical challenges in current nursing human resource allocation were identified:(1)A lack of flexibility in workforce allocation, rendering it difficult to accommodate workload fluctuations caused by reduced hospital stays and increased patient turnover under the DRG model;(2)A growing demand for specialized nursing services, yet inadequate training resources;(3)An imperfect performance evaluation system, complicating the quantitative assessment of workload, service quality, and efficiency;(4)A misalignment between nursing workload and DRG payment adjustments, leading to delayed modifications in human resource allocation;(5)Insufficient interdisciplinary collaboration between nursing staff and the medical team, negatively affecting overall service quality.

**Conclusion:**

Against the backdrop of the DRG payment model, a significant increase in nursing workload has been observed among elderly patients with severe pneumonia, while existing nursing human resource allocation remains suboptimal.

